# Serological Screening of SARS-CoV-2 Infection in Several Mammalian Species in Wilhelma Zoo, Stuttgart, Germany

**DOI:** 10.3390/pathogens13080612

**Published:** 2024-07-24

**Authors:** Jignesh Italiya, Tobias Knauf-Witzens, Annika Weigold, Jiří Černý

**Affiliations:** 1 Centre for Infectious Animal Diseases, Faculty of Tropical AgriSciences, Czech University of Life Sciences Prague, Kamýcká 129, CZ-16500 Prague, Czech Republic; italiya@ftz.czu.cz; 2 Wilhelma Zoological-Botanical Garden, 70376 Stuttgart, Germany; tiermedizin@wilhelma.de (T.K.-W.); annika.weigold@wilhelma.de (A.W.)

**Keywords:** COVID-19, zoo animals, western lowland gorillas, serological surveillance

## Abstract

Severe Acute Respiratory Syndrome Coronavirus 2 (SARS-CoV-2) affects both humans and a wide range of mammalian species globally. Between July 2022 and January 2023, fifteen blood samples were collected from twelve different animal species during veterinary examinations, as well as for health control at Wilhelma Zoo, Germany. These samples were later analyzed for the presence of SARS-CoV-2 antibodies. The serum analysis from two gorillas indicated the presence of antibodies specific to the nucleocapsid protein of SARS-CoV-2, suggesting previous infection. These gorillas were sampled in August and September 2022, during which time they exhibited symptoms such as apathy, anorexia, vomiting, and moderate diarrhea—symptoms not typically associated with COVID-19. Given that several periods of other unusual signs have been observed in the gorillas kept in Wilhelma Zoo since the onset of the COVID-19 pandemic, it remains uncertain whether these symptoms were directly related to SARS-CoV-2 infection or if these gorillas underwent clinically inapparent infection before. Nonetheless, this study underscores the importance of ongoing animal screening in zoos to better understand the spread of SARS-CoV-2 among different animal species.

## 1. Introduction

The COVID-19 pandemic, caused by Severe Acute Respiratory Syndrome Coronavirus 2 (SARS-CoV-2), is believed to have originated from bats and subsequently transmitted to humans through an intermediary animal host [1]. Globally, both symptomatic and asymptomatic cases of SARS-CoV-2 infection in animals have been reported in various zoos and among free-living wildlife [2,3]. Further, reverse zoonotic spillovers from SARS-CoV-2-infected animals to humans have also been reported [4].

Notably, wild felines have shown high susceptibility to life-threatening infections of SARS-CoV-2 [5,6,7]. The in silico analysis has identified Old World primates as highly susceptible to SARS-CoV-2 infection due to the similarity between their ACE2 receptor and that of humans [8]. Despite this expected susceptibility, natural infections have so far been observed only in gorillas, and their prevalence is relatively low compared to those in felids [3]. In captivity, SARS-CoV-2-infected animals have exhibited a range of clinical signs, such as cough, nasal discharge, and behavior changes like reduced appetite and lethargy. Captive western lowland gorillas are reported to display diverse clinical signs, including fever, coughing, and lethargy.

Zoos play a very important role in public health by enrolling standardized epidemiological surveillance of their zoological collections [9]. Investigating the SARS-CoV-2 transmission among various zoo species helps to identify potential virus reservoirs within wildlife populations. This study specifically examines the potential SARS-CoV-2 infection in symptomatic western lowland gorillas (*Gorilla gorilla gorilla*) and other asymptomatic mammals across different species by sampling and screening their sera.

## 2. Methods

A total of fifteen blood samples (5 mL, clotted) were collected from twelve distinct species across seven families between July 2022 and January 2023 by zoo veterinarians. Given that blood collection in zoo animals is known to be a stressful procedure, these samples were obtained during routine veterinary examinations rather than for the explicit purpose of testing for SARS-CoV-2 antibodies.

Serum was extracted from all fifteen samples. Samples were collected in VACUETTE^®^ TUBE 5 ml CAT Serum Separator Clot Activator (Greiner Bio-One GmbH, Frickenhausen, Germany). To obtain serum, blood was allowed to clot at room temperature for at least 40 minutes before centrifugation at 3400 RPM for ten minutes. The detection of SARS-CoV-2 antibodies in the serum specimens was performed using the ID Screen® SARS-CoV-2 Double Antigen Multi-species ELISA kit (ID VET, Montpellier, France). This double antigen enzyme-linked immunosorbent assay (ELISA) protocol aimed to identify immunoglobulin G (IgG) antibodies specific to the nucleocapsid protein of SARS-CoV-2 in the serum samples. The ELISA assays were conducted in accordance with the instructions provided by the manufacturer. The optical densities (ODs) were measured at a wavelength of 450 nm. The optical density (OD) of each sample was determined by calculating the proportion of signal to background (S/P%). According to the manufacturer’s instructions, serum samples with S/P% values exceeding 60% were categorized as positive.

## 3. Results and Discussion

Except for two samples derived from gorillas, all the other samples tested negative in the ELISA assay, as detailed in Table 1. These gorillas had not been vaccinated against SARS-CoV-2.

The western lowland gorilla population at Wilhelma Zoo consists of eleven individuals: six females and five males. The examined blood samples were collected between August and September 2022, when two female gorillas exhibited apathy and multiple gastrointestinal symptoms. Undi, a 51-year-old female, displayed clinical symptoms including anorexia, signs of fever, lameness, and stiffness in her movements. Tuana, a 17-year-old, experienced milder symptoms. Due to the severity of these clinical manifestations, the zoo veterinarians sedated the animals to perform examinations, provide medical care, and collect blood samples. It took Undi approximately six weeks and multiple treatments to fully recover. Tuana recovered within a few days.

Since the onset of the COVID-19 pandemic, gorillas at Wilhelma Zoo have displayed symptoms of SARS-CoV-2 infection on several occasions. In February 2022, Kibo (31-year-old silverback) and Milele (10-year-old female) presented with dry cough; however, the subsequent SARS-CoV-2 nasal and fecal tests yielded negative results. In April 2022, Pelu, a 4-year-old male, showed mild coughing symptoms but was not tested for SARS-CoV-2 and did not receive any medical intervention, while the other members of the group remained asymptomatic. Furthermore, alongside the two previously mentioned females, Mutasi, a 28-year-old female, was anorexic after displaying vomiting and mild diarrhea in August 2022. She recovered within a few days without treatment. Later, in September 2022, two older adult gorillas, Kibo and Kolo, a 36-year-old female, showed lameness and stiff walking but no other symptoms. Due to the mild nature of these symptoms, sedation for the diagnosis was not deemed necessary.

Only two published cases of SARS-CoV-2 infections in gorillas are described in the literature, and it is also worthwhile to report asymptomatic infections. For example, in November 2021, multiple western lowland gorillas and Asiatic lions at Rotterdam Zoo in the Netherlands exhibited fever, coughing, and lethargy, and an outbreak of COVID-19 was confirmed in both species through positive SARS-CoV-2 RT-qPCR tests. The contact tracing identified two zookeepers who tested positive for SARS-CoV-2 [10]. Nagy et al., 2022 described a COVID-19 case in the gorillas in a zoo in the Czech Republic. Clinical signs reminiscent of COVID-19 disease, such as tiredness, fatigue, dry cough, and loss of appetite, were observed. The fecal specimens showed weak positivity by RT-qPCR [11]. Unlike the literature, our study did not observe the typical respiratory signs of SARS-CoV-2 in the positive animals. Therefore, COVID-19 was not considered the primary problem, the serological testing was conducted almost half a year after the onset of the symptoms, and the contact tracing was not carried out during the sample collection period. Nevertheless, the zookeepers were advised to get vaccinated and were required to wear personal protective equipment, especially FFP2 face masks. None of the zookeepers showed any signs of SARS-CoV-2 infection.

This study has several limitations. First, further confirmatory tests were not conducted. Additionally, the presence of antibodies in gorillas does not confirm an active SARS-CoV-2 infection at the time of sampling as it may result from a past asymptomatic infection.

In conclusion, our study underscores the importance of continued surveillance for SARS-CoV-2 in zoo species, particularly given the documented instances and potential of asymptomatic transmission patterns. Further investigation is essential to fully describe the possible symptoms associated with SARS-CoV-2 infection in different species and effectively address the concerns related to the zoonotic or reverse-zoonotic transmission of SARS-CoV-2.

## Figures and Tables

**Table 1:** List of animal species used in this study: highlighted samples denote positives in ELISA assay (S/P% > 60); name of the individual animal (if given) is in brackets.

Animal Family	Animal Species	Scientific Name	Date of Sample Collection	S/P%
**Equidae**	Shetland Pony	*Equus caballus caballus*	26 July 2022	19.22679
**Suidae**	Kunekune Pig	*Sus scrofa domesticus*	1 August 2022	−7.27843
**Bovidae**	Alpine Ibex	*Capra ibex*	13 August 2022	−8.11235
**Hominidae**	**Western lowland gorilla (Tuana)**	***Gorilla gorilla gorilla***	**16 August 2022**	**719.3502**
**Bovidae**	Border Leicester	*Ovis aries*	23 August 2022	20.60776
**Bovidae**	Scimitar Oryx	*Oryx dammah*	24 August 2022	−8.57934
**Hominidae**	**Western lowland gorilla (Undi)**	***Gorilla gorilla gorilla***	**22 September 2022**	**84.59922**
**Felidae**	Asiatic Lion	*Panthera leo persica*	23 September 2022	−5.07022
**Equidae**	Shetland Pony	*Equus caballus caballus*	27 September 2022	6.911505
**Hominidae**	Bonobo	*Pan paniscus*	19 November 2022	−21.2615
**Felidae**	Snow Leopard	*Panthera uncia*	22 November 2022	−10.6208
**Bovidae**	Domestic Yak	*Bos grunniens*	23 November 2022	−17.152
**Bovidae**	Domestic Yak (Sonam)	*Bos grunniens*	30 November 2022	1.441009
**Cervidae**	Milu	*Elaphurus davidianus*	5 January 2023	8.08566
**Equidae**	Somali Wild Ass	*Equus africanus somaliensis*	17 January 2023	−10.1271

## Data Availability

The authors confirm that the data supporting the findings of this study are available within the article.

## References

[1] Cui S., Liu Y., Zhao J., Peng X., Lu G., Shi W., Pan Y., Zhang D., Yang P., Wang Q. (2022). An Updated Review on SARS-CoV-2 Infection in Animals. Viruses.

[2] Italiya J., Vacek V., Matějů P., Dering C., Celina S.S., Ndiaye A., Černý J.J.A. (2023). First detection of SARS-CoV-2 in white rhinoceros during a small-scale coronavirus surveillance in the Bandia Reserve, Senegal. Animals.

[3] Nederlof R.A., de la Garza M.A., Bakker J. (2024). Perspectives on SARS-CoV-2 Cases in Zoological Institutions. Vet. Sci..

[4] Oude Munnink B.B., Sikkema R.S., Nieuwenhuijse D.F., Molenaar R.J., Munger E., Molenkamp R., Van Der Spek A., Tolsma P., Rietveld A., Brouwer M.J.S. (2021). Transmission of SARS-CoV-2 on mink farms between humans and mink and back to humans. Science.

[5] Tewari D., Miller R., Livengood J., Wang L., Killian M.L., Bustamante F., Kessler C., Thirumalapura N., Terio K., Torchetti M.J.A. (2023). SARS-CoV-2 infection dynamics in the Pittsburgh zoo wild felids with two viral variants (Delta and alpha) during the 2021–2022 pandemic in the United States. Animals.

[6] Bartlett S.L., Koeppel K.N., Cushing A.C., Bellon H.F., Almagro V., Gyimesi Z.S., Thies T., Hård T., Denitton D., Fox K.Z.J.J.o.Z. (2023). Global retrospective review of severe acute respiratory syndrome SARS-CoV-2 infections in nondomestic felids: March 2020–february 2021. J. Zoo Wildl. Med..

[7] Italiya J., Bhavsar T., Černý J.J.V.W. (2023). Assessment and strategy development for SARS-CoV-2 screening in wildlife: A review. Vet. World.

[8] Melin A.D., Janiak M.C., Marrone F., Arora P.S., Higham J.P. (2020). Comparative ACE2 variation and primate COVID-19 risk. Commun. Biol..

[9] Van Leeuwen P., Falconer S., Veitch J., Pyott B., Hughes B., Zimmermann I., Schulte-Hostedde A. (2023). Zoos as Sentinels? A Meta-Analysis of Seroprevalence of Terrestrial Mammalian Viruses in Zoos. EcoHealth.

[10] Dusseldorp F., Bruins-van-Sonsbeek L.G., Buskermolen M., Niphuis H., Dirven M., Whelan J., Munnink B.B.O., Koopmans M., Fanoy E.B., Sikkema R.S.J.E. (2023). SARS-CoV-2 in lions, gorillas and zookeepers in the Rotterdam Zoo, the Netherlands, a One Health investigation, November 2021. Eurosurveillance.

[11] Nagy A., Stará M., Vodička R., Černíková L., Jiřincová H., Křivda V., Sedlák K.J.A.o.V. (2022). Reverse-zoonotic transmission of SARS-CoV-2 lineage alpha (B. 1.1. 7) to great apes and exotic felids in a zoo in the Czech Republic. Arch. Virol..

